# Post-matching analysis after coarsened exact matching: implications of coarsening for residual confounding and model dependence

**DOI:** 10.21203/rs.3.rs-9076964/v1

**Published:** 2026-04-03

**Authors:** Fei Wan

**Affiliations:** 1 Division of Public Health Sciences, Department of Surgery, Washington University School of Medicine

**Keywords:** Coarsened exact matching, Propensity score matching, Model dependence, Residual confounding, Bias, Curse of dimensionality

## Abstract

**Background:**

Coarsened Exact Matching (CEM) is a widely used design strategy aimed at reducing confounding in observational studies by matching treated and control units within strata of coarsened covariates. It is often promoted as a method that mimics a randomized block design, which has led many researchers to apply simple, unadjusted statistical methods—such as paired t-tests or McNemar’s test—originally developed for blocked randomized designs. However, CEM only ensures balance on the coarsened scale, and residual imbalances may remain on the original covariate scale, raising questions about the appropriateness of unadjusted analyses as the primary analytic approach.

**Methods:**

We examine the implications of this coarsening process for post-matching analysis using literature review, conceptual arguments, and simulation studies. In particular, we evaluate how within-stratum heterogeneity in the original covariates affects residual confounding and the dependence of treatment effect estimates on outcome model specification.

**Results:**

Our results show that matching on coarsened covariates can leave systematic differences between treated and control subjects within matched strata. These differences introduce residual confounding that does not disappear with increasing sample size. Simulation results further demonstrate a bias–variance trade-off induced by coarsening: fine coarsening may reduce residual confounding but can result in substantial data loss, whereas coarse binning preserves sample size at the cost of increased bias and greater reliance on outcome model specification.

**Conclusions:**

CEM should be regarded primarily as a preprocessing tool for improving covariate overlap rather than as a stand-alone solution for confounding control. Valid causal inference following CEM generally requires regression adjustment using the original, uncoarsened covariates, and unadjusted analyses of matched data may yield biased treatment effect estimates.

## Background

Coarsened exact matching (CEM) is a widely used design strategy in outcomes research for estimating the causal effects of alternative treatment options [1]. Its primary objective is to balance confounders—which are frequently unequally distributed across treatment groups in observational studies—and thereby reduce confounding bias. As a relaxed form of covariate matching, CEM temporarily coarsens covariate values into broader categories and subsequently matches units exactly within these coarsened strata, discarding any units that lack a suitable match.

CEM has been promoted as an attractive alternative to other commonly used approaches, such as propensity score matching (PSM), for several reasons [1]. First, by matching exactly on coarsened covariates, CEM guarantees balance on the coarsened variables, reducing the need for post hoc balance diagnostics on those specific variables. Second, the coarsening step allows investigators to manage the bias—variance trade-off ex ante: finer coarsening reduces imbalance but often leads to greater data loss, whereas coarser groupings retain more observations but permit greater within-stratum heterogeneity. Third, CEM bypasses the need for explicit modeling of the treatment assignment mechanism—an advantage in applied settings where specifying or justifying a propensity score model may be difficult. Finally, the resulting matched strata are transparent and clinically interpretable, fostering the perception that CEM closely resembles design-based approaches, such as stratification or blocking, typically used in randomized trials.

In many applied clinical studies, CEM has been described as an exact matching design that approximates a blocked randomized design, and CEM-matched samples are often analyzed using statistical methods traditionally applied to randomized block designs, such as paired t-tests or McNemar tests [3–9]. However, because matching is performed on coarsened rather than original covariate values, the resulting matched groups may retain substantial imbalance on the original scale [2]. As a result, the interpretation of CEM strata as randomized blocks may be misleading when systematic residual differences between treated and control subjects persist within the coarsened strata.

Despite the widespread use of CEM, the implications of residual imbalance within coarsened strata for post-matching analysis have not been fully clarified. In particular, treating CEM strata as if they arose from a randomized block design may lead to analytic choices that do not appropriately account for residual confounding. This article examines the fundamental properties of CEM and clarifies how matching on coarsened covariates differs from exact matching on the original covariates. Our goal is to provide a clearer conceptual framework for interpreting CEM-matched samples and to support the development of appropriate analytic strategies following CEM preprocessing.

## Method

### Causal Framework and Assumptions

Let T∈{0,1} denote the binary treatment indicator, where T=1 represents treatment and T=0 represents control. Let Y(1) and Y(0) denote the potential outcomes under treatment and control, respectively. Let X denote the vector of observed baseline confounders. The observed outcome is given by Y. Identification of causal effects from observational data relies on the following assumptions.

### Assumption 1 (Stable Unit Treatment Value Assumption, SUTVA).

Each unit has well-defined potential outcomes and there is no interference between units. Specifically:

1. No interference: the potential outcomes for one individual do not depend on the treatment assignments of other individuals.
2. Consistency: the observed outcome equals the potential outcome corresponding to the treatment actually received.

$$Y=TY\left(1\right)+\left(1-T\right)Y\left(0\right).$$

### Assumption 2 (Strongly ignorable treatment assignment (SITA)).

Conditional on the observed covariates X, treatment assignment is independent of the potential outcomes:

(Y(1),Y(0))⫫T∣X.

This assumption implies that all confounders affecting both treatment assignment and the outcome are captured in X.

### Assumption 3 (Positivity).

For all values of X in the support of the data, both treatment groups have positive probability:

0<P(T=1∣X)<1.

Under Assumptions 1–3, causal effects can be identified by comparing treated and control units with similar covariate values.

### Covariate Matching Designs

Exact matching attempts to directly satisfy the conditional exchangeability assumption by forming strata in which treated and control units share identical values of the covariates X. Formally, for a treated unit i and a control unit j, exact matching requires

$$X_{i}=X_{j},$$

which is equivalent to requiring that the covariate distance between the two units is zero,

$$d\left(X_{i},X_{j}\right)=\left\|X_{i}-X_{j}\right\|=0.$$

Within each stratum defined by X, comparisons between treated and control units can therefore be interpreted as unconfounded under Assumption 2.

CEM is a preprocessing method that reduces imbalance between treatment groups by temporarily coarsening covariates prior to matching [1]. The central idea is to group values of each covariate that are considered substantively similar and assign them the same value. Let C(X) denote the coarsened version of the original covariate vector X, where each covariate $X_{j}$ is divided into discrete bins $C_{j}\left(X_{j}\right)$. Coarsening can be specified by the analyst using subject-matter knowledge or determined using automatic binning rules implemented in the R MatchIt package.

Matching is then performed exactly on the coarsened covariates C(X). Formally, a treated unit iand a control unit jare considered matched if

$$C\left(X_{i}\right)=C\left(X_{j}\right),$$

or equivalently if the distance between their coarsened covariates is zero,

$$d\left(C\left(X_{i}\right),C\left(X_{j}\right)\right)=0.$$

Treated and control units with identical values of C(X) are grouped into strata,

$${𝒮}_{k}=\left\{i:C\left(X_{i}\right)=c_{k}\right\},$$

where $c_{k}$ denotes a unique combination of coarsened covariate values. Observations are retained only if both treatment groups are represented within a stratum. In this sense, CEM functions as a stratification procedure that forms many-to-many matched strata.

Within each retained stratum, weights may be assigned to account for differences in the number of treated and control subjects. Let $n_{1k}$ and $n_{0k}$ denote the numbers of treated and control units in stratum k, respectively. A common weighting scheme assigns

$$w_{i}=\left\{\begin{array}{cc} 1, & Z_{i}=1,\\ \frac{n_{1k}}{n_{0k}}, & Z_{i}=0,i\in {𝒮}_{k}, \end{array}\right.$$

so that the weighted number of control units equals the number of treated units within each stratum. Alternatively, one-to-one matching within strata may be performed to avoid weighting. After the matching step, the coarsened variables are discarded and the original (uncoarsened) covariates X are retained in the matched dataset. Subsequent estimation of treatment effects is conducted using the matched data, typically through comparisons within strata or regression modeling.

### Propensity Score

The propensity score is defined as the conditional probability of receiving treatment given covariates [10]:

$$e\left(X\right)=P\left(T=1|X\right).$$

The propensity score has two key properties.

#### Property 1 (Balancing Property).


$$T⫫X|e\left(X\right).$$

Conditional on the propensity score, the distribution of covariates X is the same for treated and control units.

#### Property 2 (Reduction of Exchangeability).

If treatment assignment is conditionally independent of the potential outcomes given X,

$$\left(Y\left(1\right),Y\left(0\right)\right)⫫T|X,$$

then it also follows that

$$\left(Y\left(1\right),Y\left(0\right)\right)⫫T|e\left(X\right).$$

Thus, adjusting for the scalar propensity score can remove confounding due to the full set of covariates X. This property implies that if SITA holds at the covariate level, it also holds at the propensity score level. That is, treatment assignment among subjects with the same propensity score can be viewed as conditionally random.

These two properties of the propensity score imply that, when treated and control units are exactly matched on the propensity score, any remaining differences in baseline covariates arise only from random variation and do not introduce residual confounding that would bias estimation of the treatment effect. Conversely, if systematic covariate differences remain after matching, this indicates that the reduction in exchangeability implied by Property 2 does not hold, suggesting the presence of residual confounding.

Formally, in PSM, units i and j are matched based on proximity of their propensity scores. Exact matching would require

$$e\left(X_{i}\right)=e\left(X_{j}\right),$$

or equivalently

$$d\left(e\left(X_{i}\right),e\left(X_{j}\right)\right)=0.$$

In practice, exact equality is rarely achievable because the propensity score is continuous. Therefore, units are typically matched by minimizing the distance

$$d\left(e\left(X_{i}\right),e\left(X_{j}\right)\right)=\left|e\left(X_{i}\right)-e\left(X_{j}\right)\right|,$$

often subject to a caliper constraint.

### Causal Estimands

We define causal estimands for our simulation studies. For continuous outcomes, we consider the treatment effect on the additive scale. The conditional average treatment effect (CATE), given a set of baseline covariates X, is defined as:

τ(X)=E[Y(1)-Y(0)∣X]

If we assume that the observed outcome Y can be described using an additive model as follows:

(1) $$E(Y|T,X)=\beta_{0}+\beta_{1}T+g(X),$$

where g(X) denotes some arbitrary function of X. Then, $\tau (X)=\beta_{1}$.

The average treatment effect (ATE) represents the expectation of the individual treatment effects across the entire population:

$$\tau_{\text{ATE}}=E[Y(1)-Y(0)]$$

By the law of iterated expectations, if the conditional treatment effect is additive and constant as defined in eq (1), these two estimands are equivalent:

$$\tau_{\text{ATE}}=E_{X}[E[Y(1)-Y(0)|X]]=E_{X}[\tau (X)]=\beta_{1}$$

In our first simulation study with continuous outcomes, we specify a data-generating process where the conditional treatment effect is constant, ensuring that both the conditional regression models and the marginal matching estimators target the same parameter $\beta_{1}$.

For binary outcomes, treatment effects are typically assessed on the multiplicative scale. The conditional odds ratio (cOR), given covariates X, is defined as:

$$\psi_{cOR}(X)=\frac{P(Y(1)=1|X)/P(Y(1)=0|X)}{P(Y(0)=1|X)/P(Y(0)=0|X)}$$

The marginal odds ratio is defined as:

$$\psi_{mOR}(X)=\frac{P(Y(1)=1)/P(Y(1)=0)}{P(Y(0)=1)/P(Y(0)=0)}$$

Unlike additive effects, the odds ratio is non-collapsible. This means the marginal (population-averaged) odds ratio is generally closer to the null than the conditional odds ratio, even in the absence of confounding [11,12]. In our simulation studies, we focus on cOR.

## Results

### Implications of coarsening for covariate balance and resulting residual differences

In an observational cohort study, subjects receive either a treatment or control at baseline and are followed until an outcome occurs. A naïve comparison between these groups is generally biased due to confounding, as treatment assignment is not randomized. Matching subjects on identical values of baseline confounders can emulate a randomized block design under SITA [13,14–18]. SITA essentially transforms an observational setting into a collection of “local experiments.” By conditioning on X, we ensure that any remaining variation in T is independent of the potential outcomes. Specifically, SITA implies that for any subjects with identical values of X, their treatment status can be considered as randomly assigned. Consequently, an exactly matched design allows each matched pair to approximate a randomized block.

However, exact matching is rarely feasible in practice due to the “curse of dimensionality”. CEM provides a practical compromise by grouping continuous covariates into categories prior to matching. For example, age might be grouped into decade-long intervals (e.g., 41–50, 51–60). Under this approach, subjects with different original values may be matched within the same coarsened stratum. As a result, treated and control subjects within these strata may differ systematically on the original covariate scale. These within-stratum differences introduce residual confounding because treatment assignment is no longer expected to behave as if randomized when conditioning only on coarsened covariates (C(X)) [2]. Consequently, matching on C(X) generally does not imply

$$\left(Y\left(1\right),Y\left(0\right)\right)⫫T|C\left(X\right),$$

Because ignorability is typically assumed conditional on the original covariates, conditioning on coarsened covariates does not generally guarantee ignorability by creating “blurred” strata where systematic associations between T and X can persist on the original scale, thereby re-introducing the very confounding it aims to eliminate. Within each coarsened stratum C(X)=C, the expected covariate imbalance between treatment groups can be expressed as

$$\delta_{c}=E(X|T=1,C(X)=c)-E(X|T=0,C(X)=c).$$

Unless the coarsening fully captures the information in X relevant to treatment assignment, $\delta_{c}$ will generally differ from zero, indicating residual imbalance in the original covariates within matched strata [2].

By contrast, PSM emulates a randomized block design through a different mechanism [11]. Under SITA, propensity score possesses the balancing score and reduction of exchangeability properties. Thus, treatment assignment may be regarded as random among subjects with the same propensity score, even if individual covariates differ. In this setting, within-pair covariate differences are not systematically related to treatment assignment. For example, a treated subject aged 41 may be matched with a control subject aged 60 if they share the same propensity score. Importantly, the age difference within such matched pairs behaves as random noises rather than systematic bias and therefore does not induce residual confounding [11–14]. As the number of matched pairs increases, the average age difference between treated and control groups converges to zero [11–14]. Unlike the random variation in PSM, the imbalance in CEM represents a non-vanishing bias that remains embedded in the matched sample regardless of sample size.

In summary, PSM achieves balance at the aggregate group level rather than the individual pair level, whereas CEM achieves balance by construction only on the coarsened scale. In practice, performing PSM with a caliper of 0.2 times the standard deviation of the logit of the propensity score can remove approximately 90% of confounding while effectively mitigating the curse of dimensionality. By contrast, the coarsening process in CEM induces systematic within-stratum imbalance on the original covariate scale. This imbalance persists both within matched pairs and, generally, at the aggregated group level. Consequently, CEM-matched data typically fail to satisfy the conditions required to interpret matched strata as randomized blocks. This fundamental divergence distinguishes the analytic requirements of CEM from those of propensity score or exact matching.

### Coarsening increases model dependence

Current clinical literature frequently refers to CEM as an approximation of randomized designs, often applying unadjusted analyses such as the paired t-test or McNemar’s test [4–8]. In randomized block trials, common analytic approaches include the paired t-test for continuous outcomes and the Cochran–Mantel–Haenszel (CMH) test—or McNemar’s test in one to one matching designs—for binary outcomes. These unadjusted analyses implicitly assume no confounding. While unadjusted analyses are generally appropriate for exact matching and are reasonable after well-executed PSM, as both designs approximate block-randomized trials under SITA [10,15–18]. Applying them to CEM-matched data is generally inappropriate. Because coarsening introduces residual confounding on the original covariate scale, the matched sample fails to replicate the properties of a blocked randomized experiment [2]. Consequently, statistical methods reserved for randomized designs cannot be directly applied. Instead, valid analysis of CEM-matched data requires regression adjustment using the original, uncoarsened covariates to mitigate this residual bias [2].

Understanding this requirement requires a clear grasp of the connection between model dependence and confounding. Model dependence refers to the sensitivity of estimated treatment effects to seemingly minor changes in model specification. As shown in Table 1 (Column 2), conventional regression analyses applied to unmatched observational data are highly model-dependent: treatment effect estimates vary substantially across models, and significant bias arises when the outcome model is misspecified (e.g., omitting quadratic terms or interactions). In such settings, unbiased estimation requires a correctly specified regression model—a goal rarely achievable in practice given high-dimensional confounding and limited sample sizes.

By contrast, matching designs can substantially reduce model dependence. As illustrated in Table 1 (Columns 3 and 4), we fitted seven regression models to matched datasets generated using exact matching and PSM. Across both designs, the average estimated treatment effects remained close to the true effect (1.5), indicating that even misspecified models yield approximately unbiased estimates. This highlights a key advantage: exact matching and PSM reduce the reliance on correct model specification, whereas unmatched regression adjustment remains inherently fragile. CEM, however, generally does not share this robust property. Because coarsening induces systematic residual confounding—and more aggressive coarsening exacerbates this issue—regression adjustment remains essential after matching. This necessity reintroduces sensitivity to model specification. Consequently, CEM increases model dependence relative to exact matching and PSM, undermining one of the principal motivations for matching as a design-based approach to confounding control. It is also worth noting that matching does not universally reduce model dependence. For example, in case–control studies, matching on confounders can increase the complexity of the relationship between the outcome and the matched variables, thereby increasing model dependence relative to unmatched designs [19–21].

To illustrate the connection between coarsening and model dependence, we conducted a simulation study with a binary treatment T, a binary outcome Y, a continuous confounder $X_{1}$, and a binary confounder $X_{2}$. After generating the data, we applied CEM by categorizing $X_{1}$ into K equal-width bins (K=5 to 50). Within each coarsened stratum, treated and control subjects were randomly paired to form matched pairs. For each level of coarsening, we estimated treatment effects using three approaches: (i) an **unadjusted analysis**, which involved computing the McNemar odds ratio without further covariate adjustment. This serves as a baseline misspecified model; (ii) an **adjusted but misspecified analysis**, estimating the cOR from a conditional logistic regression adjusting only for $X_{1}\left[\text{cLogit}\left(T,X_{1}\right)\right]$; and (iii) an **adjusted analysis with a correctly specified model**, estimating the OR from a conditional logistic regression adjusting for $X_{1},X_{1}^{2}$, and the interaction $X_{1}\times X_{2}\left[\text{cLogit}\left(T,X_{1},X_{1}^{2},X_{1}\times X_{2}\right)\right]$. As summarized in Table 2, the unadjusted McNemar odds ratio exhibits the largest bias, which intensifies as coarsening becomes more aggressive (fewer bins). The misspecified adjusted model, $\text{cLogit}\left(T,X_{1}\right)$, reduces bias but remains sensitive to the degree of coarsening. Only the correctly specified adjusted model yields stable, nearly unbiased estimates across all coarsening levels.

A common motivation for using CEM is the desire for a “model-free” analysis that avoids the complexities of propensity score specification. However, our results show that CEM primarily shifts the modeling burden from the treatment assignment to the outcome. Because coarsening leaves residual confounding, the final treatment effect estimate remains highly sensitive to the post-matching regression model. As shown in Table 2, unadjusted methods like McNemar’s test fail to recover the true effect as coarsening becomes more aggressive, with the cOR collapsing from 1.48 to 0.397 as the number of bins decreases. Thus, the perceived simplicity of CEM may come at the cost of increased model dependence—undermining the very advantage it seeks to provide.

### Coarsening choice is a bias-variance trade-off

A central feature of CEM is that investigators must choose the degree of coarsening for each covariate prior to matching. This choice directly determines the bias–variance trade-off of the resulting analysis and has significant implications for both statistical efficiency and causal validity. With finer coarsening—such as the auto-coarsening algorithms—CEM more closely approximates exact matching on the original covariate scale. In principle, finer coarsening reduces residual confounding by limiting within-stratum heterogeneity. In practice, however, as the dimensionality of the covariate set increases, fine coarsening rapidly exacerbates the “curse of dimensionality.” The number of potential matched strata grows combinatorially, leading to severe data loss and very small, matched samples. Consequently, treatment effect estimates become highly variable and unstable, and statistical inference may be driven by a small, potentially unrepresentative subset of the original cohort [2, 22,23].

At the other extreme, coarser coarsening increases the size of the matched sample and improves the stability of effect estimate, but at the cost of increased residual confounding. Broad coarsening permits substantial within-stratum imbalance on the original covariate scale, inducing systematic differences between treated and control groups that persist after matching. In this setting, unadjusted analyses are no longer valid, and regression adjustment using the original, uncoarsened covariates becomes essential. Consequently, effect estimation becomes sensitive to correct model specification, reintroducing model dependence and undermining the design-based appeal of matching [2].

Importantly, this trade-off is not merely statistical but conceptual. Fine coarsening prioritizes bias reduction at the expense of variance, whereas coarse coarsening prioritizes variance reduction at the expense of bias. Unlike propensity score–based methods—where the balancing property holds under SITA—CEM does not provide a principled criterion for selecting an “optimal” coarsening scheme that simultaneously minimizes bias and variance. Different coarsening choices may, therefore, yield divergent effect estimates when using misspecified models (such as unadjusted analyses), complicating both interpretation and reproducibility.

Moreover, coarsening decisions are often data-dependent and analyst-driven, introducing an additional layer of subjectivity. Seemingly minor changes in bin width or category cut points can alter the matched sample composition, the degree of residual confounding, and the extent of model dependence in subsequent analyses. In applied research, these choices are rarely justified or systematically examined, yet they can materially affect causal conclusions.

The researcher using CEM thus faces a fundamental dilemma: The paradox of precision. To eliminate residual confounding, one must employ fine coarsening; yet, in high-dimensional settings, this triggers a loss of matches for the majority of treated units. To maintain a representative sample size, researchers often resort to “coarse” coarsening. This decision—frequently made without a principled objective—preserves the sample size at the expense of introducing systematic bias. Without transparent justification for these binning choices, causal conclusions become sensitive to subjective analyst decisions, ultimately complicating the reproducibility of clinical findings.

Taken together, the choice of coarsening in CEM represents a fundamental bias–variance trade-off with no universally optimal solution. While CEM can be a useful preprocessing tool, its performance depends critically on coarsening decisions that directly influence data loss, residual confounding, and model dependence. Recognizing and explicitly addressing this trade-off is essential for valid causal inference using CEM.

## Discussion

A key but often overlooked feature of coarsened exact matching is that exact balance is achieved only on the coarsened covariates, not on the original covariate scale. Consequently, individuals within the same CEM stratum may still differ substantially in their original covariate values. When the outcome depends on the original covariates rather than their coarsened versions, such within-stratum heterogeneity induces residual confounding that is not eliminated by the matching step alone. This residual imbalance arises from the coarsening itself and therefore does not disappear with increasing sample size. CEM can only fully emulate a randomized block design in the exceptional case where coarsening entails no information loss and the coarsened categories are equivalent to the original variables; in practice, this condition is rarely met.

As a result, investigators face a stark trade-off: fine coarsening may reduce residual confounding but often results in substantial data loss and unstable effect estimates in high-dimensional settings, occasionally rendering matching infeasible [2,22,23]. In contrast, coarse coarsening yields larger matched samples at the expense of increased residual confounding, biased effect estimates, and heightened dependence on post-matching modeling assumptions. Consequently, in practical applications involving numerous matching variables, CEM may perform less optimally than PSM, and unadjusted analyses should not serve as the primary analytic approach following its implementation [2, 22, 23].

## Conclusion

In conclusion, CEM should be viewed as a powerful preprocessing tool for improving covariate overlap, rather than a stand-alone solution for confounding control. To ensure the validity of causal inferences, we recommend that applied studies:

1. Avoid unadjusted analyses (e.g., paired t-tests or McNemar’s tests) as the primary reported result, as these fail to account for within-stratum heterogeneity.
2. Prioritize regression adjustment using original, uncoarsened covariates in the post-matching model to mitigate residual confounding.
3. Report the sensitivity of results to various coarsening schemes to demonstrate the robustness of the findings to different binning decisions.

## Figures and Tables

**Table 1:** Averaged treatment effect estimates from seven regression models in unmatched, exact matching, and PSM designs for a simple simulation study^§^

No	Regression Model	Average Treatment Effect Estimate in Unmatched Design^1^	Average Treatment Effect Estimate in Exact matching Design^2^	Average Treatment Effect Estimate in PSM Design^3^
1	M(A)	5.346	1.503	1.499
2	$M\left(A,X_{1}\right)$	2.126	1.503	1.499
3	$M\left(A,X_{2}\right)$	3.660	1.503	1.499
4	$M\left(A,X_{1},X_{2}\right)$	0.363	1.503	1.499
5	$M\left(A,X_{1},X_{2},X_{1}X_{2}\right)$	1.120	1.503	1.499
6	$M\left(A,X_{1},X_{1}^{2},X_{2}\right)$	0.739	1.503	1.499
7	$M\left(A,X_{1},X_{1}^{2},X_{2},X_{1}X_{2}\right)$	1.498	1.503	1.499

§ In this simple simulation study, we generate 1,000 data sets (each with a sample size n=10000) with one discrete confounder ($X_{1}$, 0–10), one binary confounder ($X_{2}$), a binary treatment (A), and a continuous outcome (Y). The correct outcome model (Model 7) includes a quadratic term for $X_{1}$ and an $X_{1}X_{2}$ interaction. The true treatment effect is 1.5.

1 This column reports the average treatment effect estimates from seven models across 1,000 unmatched original datasets. Values close to 1.5 indicate approximately unbiased estimation. M(A) represents the unadjusted model, which includes only the treatment variable.

2 This column reports the average treatment effect estimates from seven models across 1,000 exact-matched datasets.

3 This column reports the average treatment effect estimates from seven models across 1,000 PSM-matched datasets.

**Table 2.** Comparison of three methods for analyzing CEM designs across varying coarsening levels2

Number of coarsened groups for $X_{1}$	Conditional Odds Ratio Estimate True cOR = 1.5		
	McNemar Method-No X	$\text{cLogit}\;(T,X_{1})$	$\text{cLogit}\;\left(T,X_{1},X_{1}^{2},X_{1}X_{2}\right)$
50	1.480	1.490	1.500
30	1.440	1.480	1.500
20	1.340	1.440	1.500
10	0.985	1.180	1.510
5	0.397	0.848	1.510

For each dataset (n = 10,000), we generated covariates $X_{1}\sim N(0,10)$ and $X_{2}\sim \text{Bernoulli}(0.5)$. Treatment was simulated from the logistic model $\text{logit}\;P(T=1)=\gamma_{0}+0.15X_{1}-0.05X_{1}^{2}+0.1X_{2}+0.08X_{1}X_{2}$. The outcome was generated using $\text{logit}\;P\left(Y=1\right)=\alpha_{0}+\text{log}\left(1.5\right)T-0.1X_{1}+0.08X_{1}^{2}-0.15X_{2}-0.02X_{1}X_{2}$, so the true causal odds ratio for Twas 1.5, and both covariates influenced treatment and outcome through nonlinear (quadratic and interaction) terms. The intercepts $\gamma_{0}$ and $\alpha_{0}$ were determined by rootfinding to yield approximately 30% treated subjects and 40% events. OR estimates were averaged over 5,000 replications.

## Data Availability

No datasets are used in this study
